# Role of Scx^+^/Sox9^+^ cells as potential progenitor cells for postnatal supraspinatus enthesis formation and healing after injury in mice

**DOI:** 10.1371/journal.pone.0242286

**Published:** 2020-12-01

**Authors:** Katsumasa Ideo, Takuya Tokunaga, Chisa Shukunami, Aki Takimoto, Yuki Yoshimoto, Ryuji Yonemitsu, Tatsuki Karasugi, Hiroshi Mizuta, Yuji Hiraki, Takeshi Miyamoto

**Affiliations:** 1 Department of Orthopaedic Surgery, Faculty of Life Sciences, Kumamoto University, Kumamoto, Japan; 2 Department of Molecular Biology and Biochemistry, Graduate School of Biomedical and Health Sciences, Hiroshima University, Hiroshima, Japan; 3 Laboratory of Cellular Differentiation, Institute for Frontier Life and Medical Sciences, Kyoto University, Kyoto, Japan; Università degli Studi della Campania, ITALY

## Abstract

A multipotent cell population co-expressing a basic-helix-loop-helix transcription factor scleraxis (Scx) and SRY-box 9 (Sox9) has been shown to contribute to the establishment of entheses (tendon attachment sites) during mouse embryonic development. The present study aimed to investigate the involvement of Scx^+^/Sox9^+^ cells in the postnatal formation of fibrocartilaginous entheses and in the healing process after injury, using *ScxGFP* transgenic mice. We demonstrate that Scx^+^/Sox9^+^ cells are localized in layers at the insertion site during the postnatal formation of fibrocartilaginous entheses of supraspinatus tendon until postnatal 3 weeks. Further, these cells were rarely seen at postnatal 6 weeks, when mature fibrocartilaginous entheses were formed. Furthermore, we investigated the involvement of Scx^+^/Sox9^+^ cells in the healing process after supraspinatus tendon enthesis injury, comparing the responses of 20- and 3-week-old mice. In the healing process of 20-week-old mice with disorganized fibrovascular tissue in response to injury, a small number of Scx^+^/Sox9^+^ cells transiently appeared from 1 week after injury, but they were rarely seen at 4 weeks after injury. Meanwhile, in 3-week-old mice, a thin layer of fibrocartilaginous tissue with calcification was formed at healing enthesis at 4 weeks after injury. From 1 to 2 weeks after injury, more Scx^+^/Sox9^+^ cells, widely distributed at the injured site, were seen compared with the 20-week-old mice. At 4 weeks after injury, these cells were located near the surface of the recreated fibrocartilaginous layer. This spatiotemporal localization pattern of Scx^+^/Sox9^+^ cells at the injured enthesis in our 3-week-old mouse model was similar to that in postnatal fibrocartilaginous enthesis formation. These findings indicate that Scx^+^/Sox9^+^ cells may have a role as entheseal progenitor-like cells during postnatal maturation of fibrocartilaginous entheses and healing after injury in a manner similar to that seen in embryonic development.

## Introduction

Entheses, the attachment sites between tendons and bone, are classified into two types according to their histological appearance at the tendon-bone interface: fibrous entheses, which typically insert into the metaphyses or diaphyses of long bones and include a periosteal component, and fibrocartilaginous entheses, which typically insert into epiphyses or bone ridges, such as the rotator cuff tendon or Achilles tendon enthesis [1, 2].

Fibrocartilaginous entheses can bear stress concentrations that arise between the tendon and bone, and prevent injury and failure using structural gradients, viz. tendon, uncalcified fibrocartilage, and calcified fibrocartilage [3–5]. Moreover, the stress concentrations at enthesis occasionally cause injury, such as rotator cuff tears [6, 7]. It has been demonstrated that the regeneration of functional fibrocartilaginous enthesis is difficult after injury or surgical tendon-bone repair because of poor healing capability, as demonstrated by the weak fibrovascular scar tissue formed between tendon and bone [8–10]. It was also shown that the repair site sometimes fails postoperatively [11]. Thus, understanding of the natural development of fibrocartilaginous enthesis, which occurs postnatally [12], and of the healing process after injury, are crucial for developing therapeutic strategies to reconstruct functional fibrocartilaginous enthesis in order to improve clinical outcomes [13, 14].

Recently, it was demonstrated that a specific multipotent cell population co-expressing scleraxis (Scx) [15, 16] and SRY-box 9 (Sox9) [17], giving rise to tenocytes, ligamentocytes, and chondrocytes, transiently appears between the adjacent Achilles tendon and cartilage primordia during a mouse embryonic development [18]. Additionally, conditional inactivation of Sox9 in Scx^+^/Sox9^+^ cells using a *ScxCre;Sox9*^*flox/flox*^ mouse resulted in the defective formation of Achilles tendon enthesis. Therefore, Scx^+^/Sox9^+^ cells are crucial in the formation of enthesis [18, 19]. The Scx^+^/Sox9^+^ progenitors may be involved in the postnatal formation of fibrocartilaginous enthesis and healing after injury, but this has not been confirmed to date.

In the present study, we investigated the existence of Scx^+^/Sox9^+^ cells during the postnatal formation of fibrocartilaginous entheses of supraspinatus tendon using *ScxGFP* transgenic (Tg) mice. We also investigated whether Scx^+^/Sox9^+^ cells are involved in the spontaneous healing process after injury to fibrocartilaginous enthesis of supraspinatus tendon in 20- and 3-week-old mice.

## Materials and methods

### Animals and healing model of supraspinatus tendon enthesis after injury

**Fig 1:**
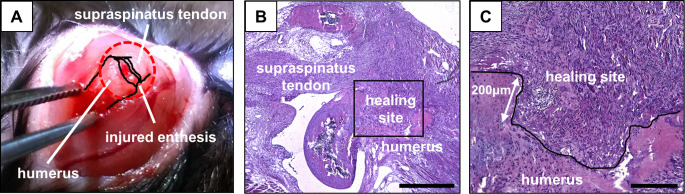
A healing model of the supraspinatus tendon enthesis. (A) An enthesis injury was made in the left shoulder of *ScxGFP* transgenic mice. (B and C) The section stained with hematoxylin and eosin (HE) under 40-fold magnification (B) and 100-fold magnification (C) at 2 weeks after injury. Bar = 500 μm in B and 200 μm in C.

We used *ScxGFP* Tg mice, which have been reported previously [20]. To investigate the localization of the Scx^+^/Sox9^+^ cells during the postnatal formation of fibrocartilaginous enthesis of supraspinatus tendon, neonatal and 3-, 6-, and 20-week-old mice were euthanized and both the shoulders were harvested (six mice per time point). In addition, to evaluate the involvement of Scx^+^/Sox9^+^ cells in the healing process after injury to the rotator cuff enthesis, we made a healing model of supraspinatus tendon entheses in 20- and 3-week-old mice, as described previously [21]. In brief, after administering general anesthesia with an intraperitoneal injection of 0.3 mg/kg medetomidine hydrochloride, 4 mg/kg midazolam, and 5 mg/kg butorphanol, 5-mm skin and deltoid muscle incisions were made on the left shoulder, and the insertion of the supraspinatus tendon was sharply detached; a cylindrical defect was created into the bone marrow at the supraspinatus attachment site using a high-speed bur (1 mm-diameter), and the fibrocartilaginous layer at the bone surface was removed (Fig 1A). The skin and deltoid incisions were closed in layers with 5–0 nylon sutures. For the sham procedure on the right shoulder, skin and deltoid incisions were made to expose the supraspinatus tendon, and were closed in the same way. At 3 days, and 1, 2, and 4 weeks after injury, the mice were euthanized (six mice per time point in each group). All experimental procedures were approved by the Animal Studies Committee and the Institutional Animal Care and Use Committee at Kumamoto University, Japan.

### Histology

As described previously [18], samples were fixed with 4% PFA/PBS at 4°C for 3 hours, treated with 20% sucrose in PBS, and frozen in SCEM-L1 compound (Section-lab, Hiroshima, Japan). Thereafter, the samples were cryosectioned to a thickness of 5 μm on a Leica CM3050 S cryostat (Leica, Wetzlar, Germany). For evaluating the histomorphology of the entheses, sections were stained with 0.05% toluidine blue (TB) solution (pH 4, Wako, Osaka, Japan) for TB staining, and with an alkaline phosphatase (ALP) staining kit (Cosmobio, Tokyo, Japan) and alizarin red S (Sigma-Aldrich, Tokyo, Japan) for ALP/alizarin red S staining, respectively. Each section was observed under a BZ-X700 microscope (Keyence, Osaka, Japan) and digital images were captured.

### Imaging of the second harmonic generation for collagen

The orientation of collagen fibers in entheses was determined by monitoring the second harmonic generation (SHG) for collagen using a multiphoton microscope (FV1000-MPE, Olympus, Tokyo, Japan), as previously described [22]. The SHG for collagen was visualized at an excitation wavelength of 950 nm using a bandpass filter of 465–485 nm.

### Immunohistochemistry

After decalcification with ethylenediaminetetraacetic acid (EDTA, 0.25 mol/L)/PBS for 60 minutes at 25°C, 5-μm thick sections were treated with hyaluronidase (1000 U/mL) for 30 minutes at 37°C, incubated overnight at 4°C with primary antibodies against GFP (diluted 1:1000; Nakarai, Kyoto, Japan), Sox9 (1:600; Millipore, Billerica, USA), and Ki-67 (1:200; Abcam, Cambridge, UK) diluted with 2% skimmed milk in PBS. The sections were then incubated with secondary antibody conjugated to Alexa Fluor 488 or 594 (Life Technologies Corporation, California, USA) for 120 minutes at 25°C. Slides were mounted and counterstained with Vectashield mounting medium for fluorescence with DAPI (Vector Laboratories, Burlingame, USA), and were imaged under a BZ-X700 microscope. The percentages of Scx-, Sox9- and Ki-67-positive cells were calculated by dividing the number of positive cells for each marker by the total number of DAPI-positive cells in square areas (100 μm × 100 μm) randomly selected from the enthesis area under 200-fold magnification (Fig 1B and 1C); the average percentage of three areas from each slide was recorded.

### Statistical analyses

All results are expressed as means ± standard deviation (SD). Statistical analyses were performed using EZR software (Jichi Medical University, Saitama, Japan) [23]. Statistical significance of comparisons between the 20- and 3-week-old mice at each time point was estimated using the Mann-Whitney U test. Differences in the measurements within groups over time were estimated using the Kruskal-Wallis test, followed by the post hoc Steel-Dwass test for multiple comparisons. Statistical significance was set at p<0.05.

## Results

### Postnatal formation of fibrocartilaginous enthesis of the supraspinatus tendon

**Fig 2:**
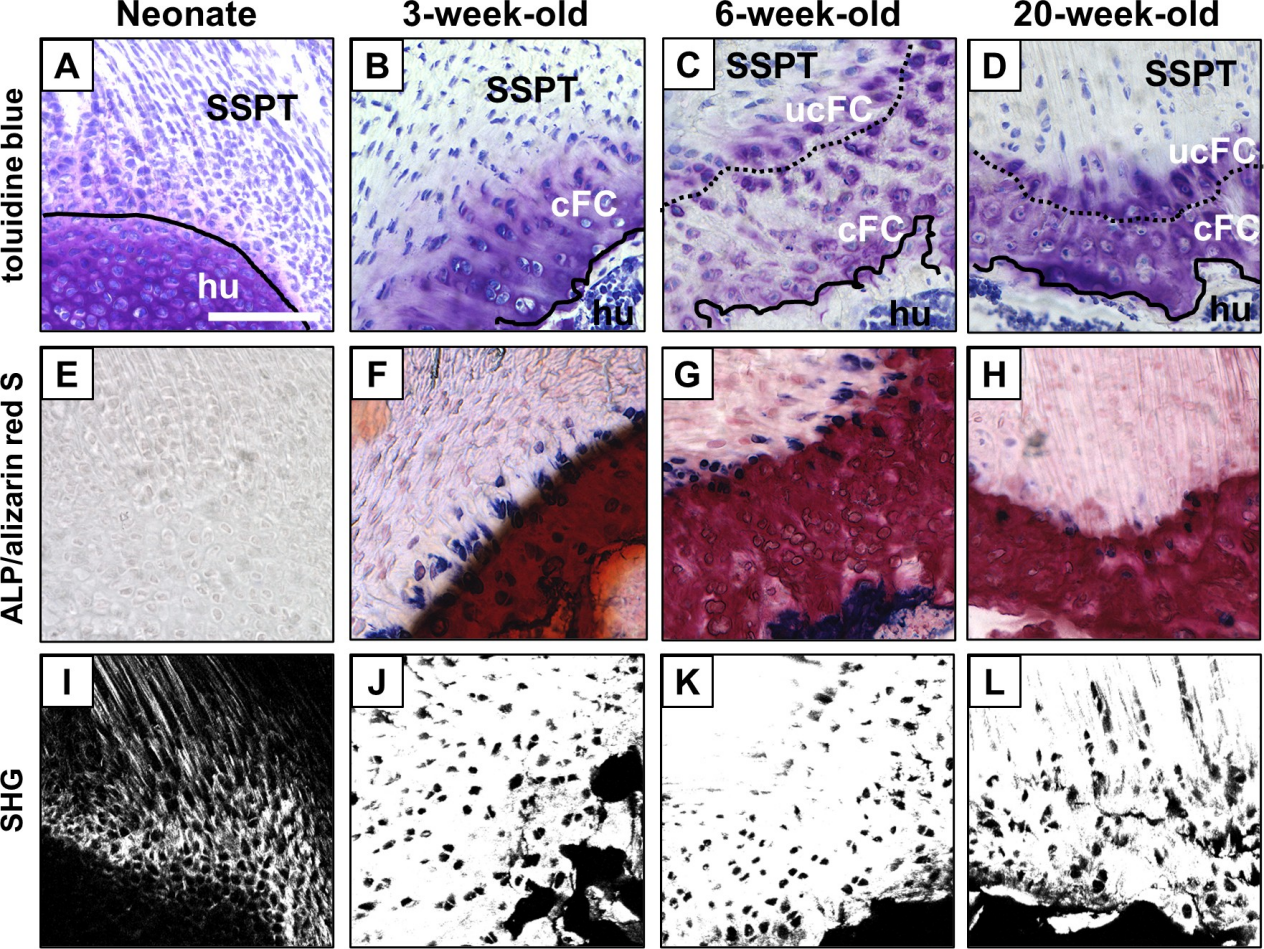
Postnatal formation of the supraspinatus tendon enthesis in mice. (A-H) Microscopic images of the supraspinatus tendon enthesis during postnatal development after staining with toluidine blue (A-D) and alkaline phosphatase (ALP)/alizarin red S (E-H). (I-L) Image of the second harmonic generation (SHG, white) of the tendon attachment sites. SSPT, supraspinatus tendon; hu, humerus; cFC, calcified fibrocartilage; ucFC, uncalcified fibrocartilage. Solid lines and dotted lines highlight the surface of humeral bone and the interface between cFC and ucFC, respectively. Scale bar = 100 μm.

In the neonate, the hyper-cellular supraspinatus tendon directly attached to the cartilage of the humeral head. No fibrocartilaginous tissue, calcified tissue, or ALP-positive area was observed at the attachment site (Fig 2A and 2E). At 3-weeks of age, a thin layer of fibrocartilaginous tissue without tidemark was observed between the supraspinatus tendon and the bone surface of the humeral head (Fig 2B), and some ALP-positive cells were detected near the surface of the fibrocartilage layer (Fig 2F). At 6-weeks of age, the mature enthesis consisting of four zones, i.e., tendon, uncalcified fibrocartilage, calcified fibrocartilage, and bone, was observed (Fig 2C). Additionally, an ALP-positive area was localized near the surface of the fibrocartilage layer similar to that in the 3-week-old mice (Fig 2G). At 20-weeks of age, histological findings of the fibrocartilaginous enthesis were similar to those in the 6-week-old mice (Fig 2D). The ALP-positive area decreased, and a few ALP-positive cells were observed at the enthesis (Fig 2H). The SHG signal for collagen at the enthesis, indicating the orientation of the collagen fibers, increased from neonate to 3-weeks of age (Fig 2I–2L).

### Scx^+^/Sox9^+^ cells participate in postnatal formation of fibrocartilaginous enthesis

**Fig 3:**
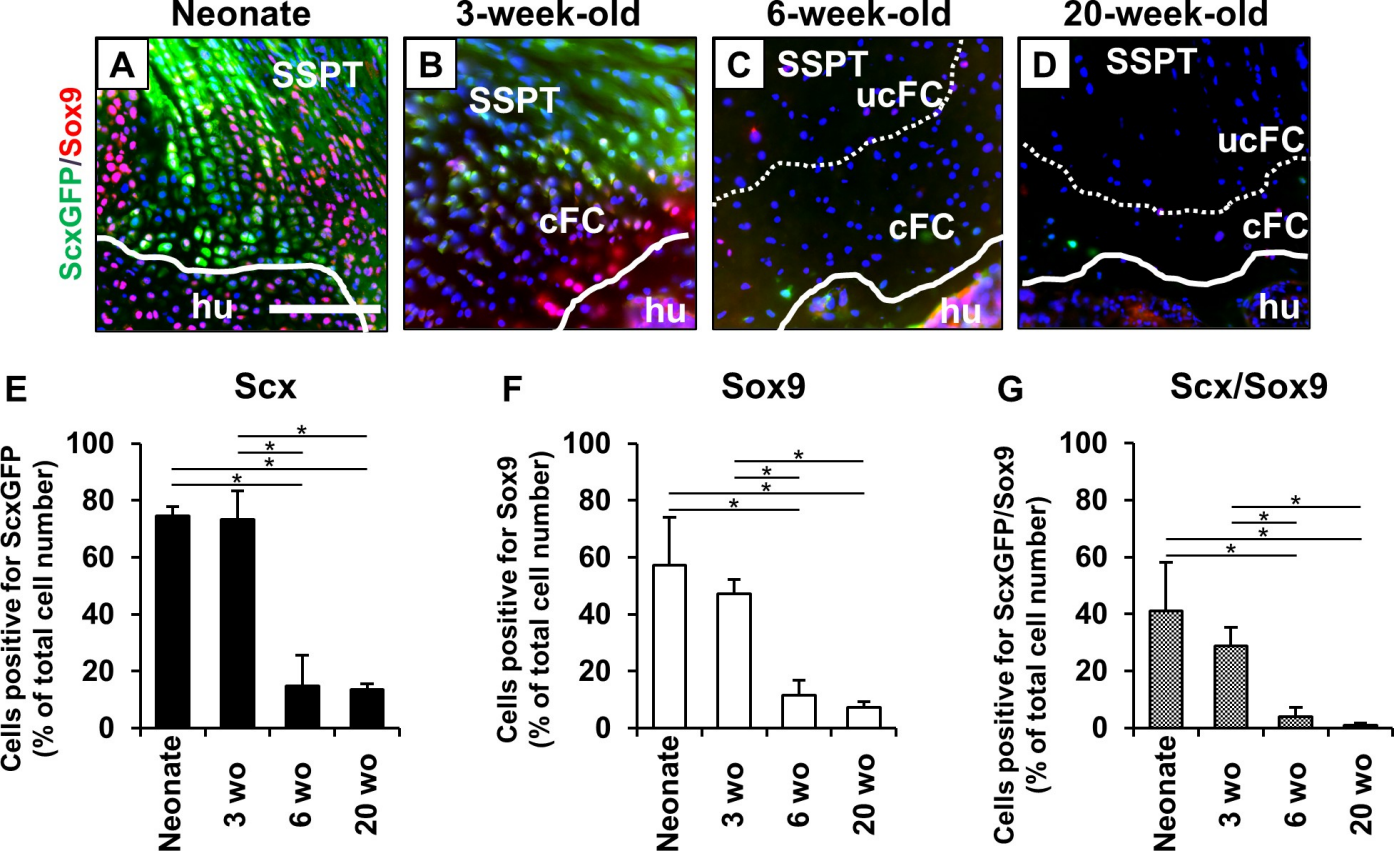
Scx^+^/Sox9^+^ cells participate in postnatal formation of fibrocartilaginous enthesis. (A-D) Immunostaining of ScxGFP (green), Sox9 (red) and DAPI (blue). SSPT, supraspinatus tendon; hu, humerus; cFC, calcified fibrocartilage; ucFC, uncalcified fibrocartilage. Solid lines and dotted lines highlight the surface of humeral bone and interface between cFC and ucFC, respectively. Scale bar = 100 μm. (E-G) The number of cells positive for ScxGFP (E), Sox9 (F), and ScxGFP/Sox9 (G) was normalized to the total number of nuclei with DAPI staining in the supraspinatus tendon enthesis in neonates, and 3-, 6-, and 20-week-old mice. All values represent means ± SD. N = 6 for each time point. *p < 0.05.

In a neonatal enthesis, many oval-, spindle-shaped Scx^+^/Sox9^+^ cells were observed from the tendon proper to the attachment site (Fig 3A). At 3-weeks of age, the Scx^+^/Sox9^+^ cells were obviously decreased compared to the neonate, and were distributed in layers, mainly observed from the surface of the fibrocartilage layer to the tendon end (Fig 3B). At 6- and 20-weeks of age, a few Scx^+^ and Sox9^+^ cells were observed; the Scx^+^/Sox9^+^ cells were rarely seen at the enthesis (Fig 3C and 3D). The percentages of Scx^+^/Sox9^+^ cells at the enthesis decreased over time (Fig 3E–3G). They were significantly higher in the neonate (p = 0.02 between the neonate and 6-weeks of age; p = 0.02 between the neonate and 20-weeks of age) and at 3 weeks of age (p = 0.02 between 3- and 6-weeks of age; p = 0.02 between 3- and 20-weeks of age) than in the 6- and 20-week-old mice.

### Healing process after supraspinatus tendon enthesis injury in 20-week-old mice

**Fig 4:**
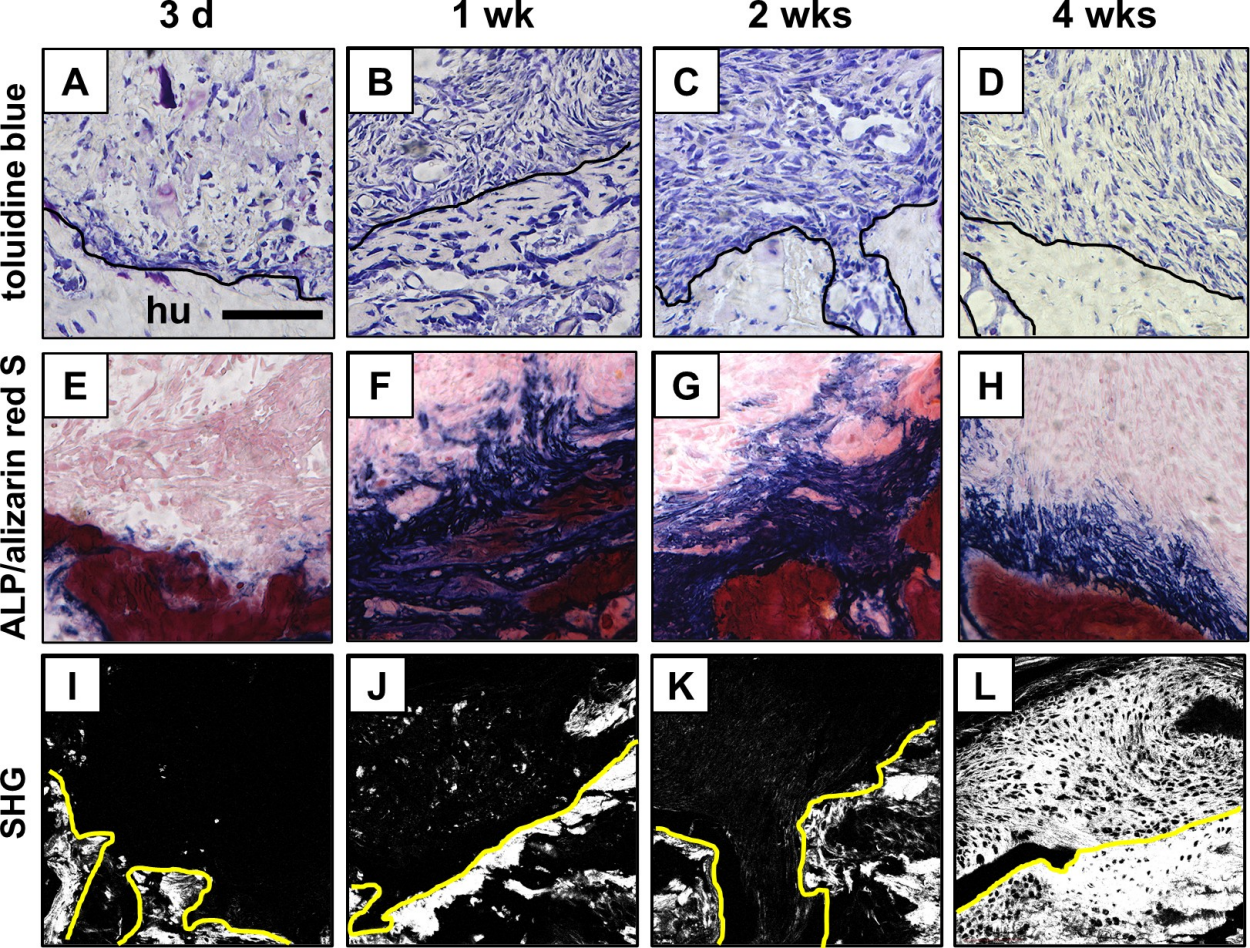
Healing process after supraspinatus tendon enthesis injury in 20-week-old mice. (A-L) Microscopic images of the tendon-bone attachment sites at 3 days (d), and 1, 2, and 4 weeks (wks) after injury in 20-week-old (wo) mice, stained with toluidine blue (A-D), and alkaline phosphatase (ALP)/alizarin red S (E-H). Image of the second harmonic generation (SHG, white, I-L). hu, humerus. Solid lines highlight the surface of the humeral bone. Scale bar = 100 μm.

At 3 days after injury, few cells with small, spherical nuclei were observed within the loose fibrous reparative tissues between the torn supraspinatus tendon and the bone (Fig 4A). Near the bone surface, small ALP-positive areas were detected within the reparative tissues (Fig 4E). At 1 and 2 weeks after injury, hyper-vascular and hyper-cellular granulation tissues were observed between the tendon and bone surface (Fig 4B and 4C), and the ALP-positive area near the bone surface enlarged to the reparative tissue when compared with the situation 3 days after injury (Fig 4F and 4G). At 4 weeks after injury, the cellularity and vascularity at the reparative tissues decreased compared with that at 1 and 2 weeks after injury (Fig 4D); there was no obvious change in the ALP-positive area near the bone surface (Fig 4H). By 4 weeks after injury, the native fibrocartilaginous tissue with tidemark was not recreated at the healing site in any of the specimens (Fig 4A–4D and 4H). The orientation of collagen fibers at the healing site improved over time as indicated by an enlargement of the area, which revealed a high signal of SHG for collagen (Fig 4I–4L).

### Healing process after supraspinatus tendon enthesis injury in 3-week-old mice

**Fig 5:**
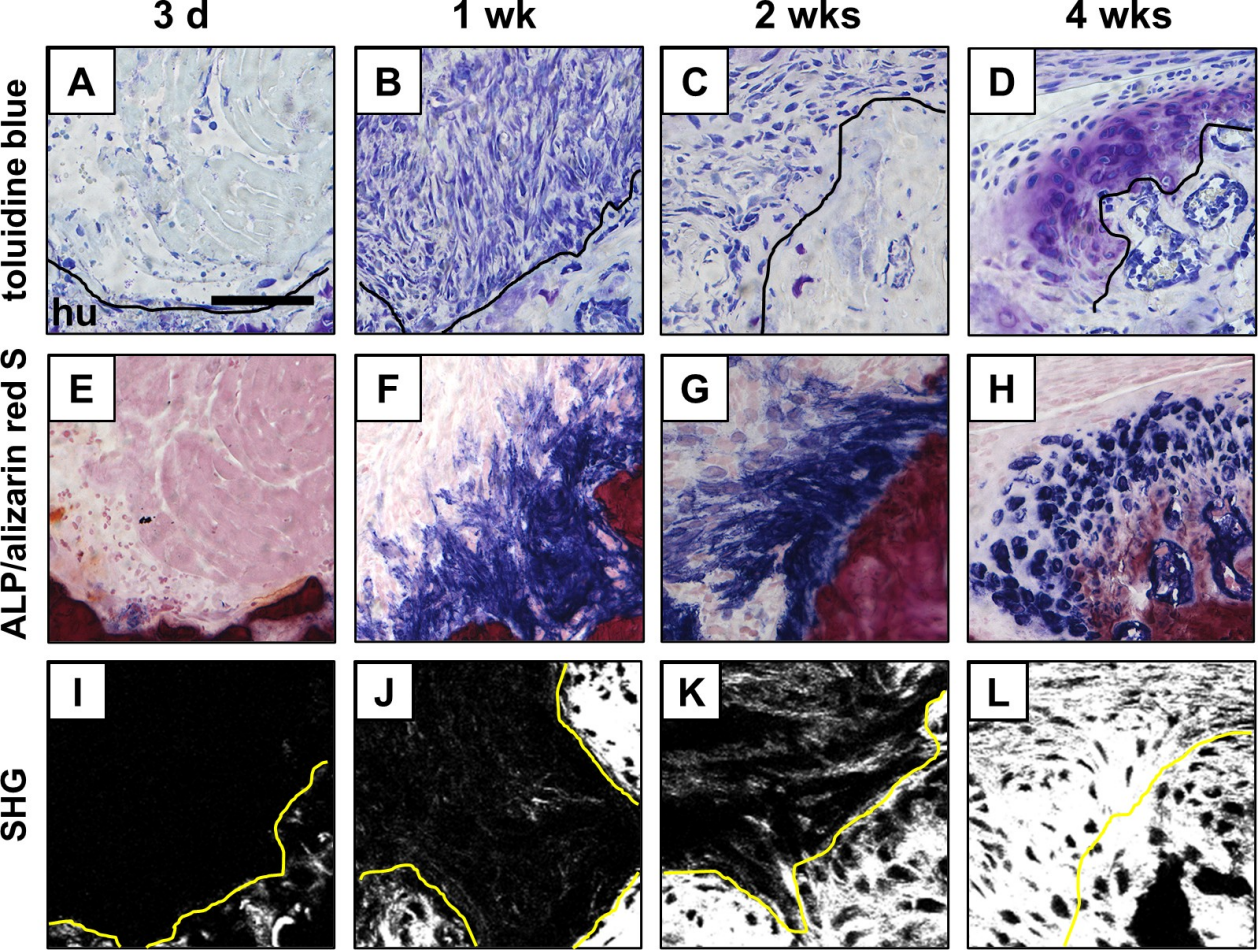
Healing process after supraspinatus tendon enthesis injury in 3-week-old mice. (A-L) Microscopic images of the tendon-bone attachment sites at 3 days (d), and 1, 2, and 4 weeks (wks) after injury in 3-week-old (wo) mice, stained with toluidine blue (A-D), and alkaline phosphatase (ALP)/alizarin red S (E-H). Image of the second harmonic generation (SHG, white, I-L). hu, humerus. Solid lines highlight the surface of humeral bone. Scale bar = 100 μm.

At 3 days after injury, a few cells with small, spherical nucleus filled the defect between the torn supraspinatus tendon and the bone, and small ALP-positive areas were detected at the reparative tissue near the bone surface as in 20-week-old mice (Fig 5A and 5E). At 1 and 2 weeks after injury, hyper-vascular and hyper-cellular granulations were observed at the reparative tissue (Fig 5B and 5C), and the enlarged ALP-positive area was observed near the bone surface as in 20-week-old mice (Fig 5F and 5G). At 4 weeks after injury, in contrast to that in 20-week-old mice, fibrocartilaginous tissue with calcified and ALP-positive area was formed on the bone surface at the reparative site in the majority of specimens, although there was no organized 4-layer structure as observed in normal enthesis (Fig 5D and 5H). The SHG signal intensity for the orientation of collagen fibers improved over time (Fig 5I–5L).

### Localization of Scx^+^/Sox9^+^ cells during enthesis healing process after injury in 20- and 3-week-old mice

**Fig 6:**
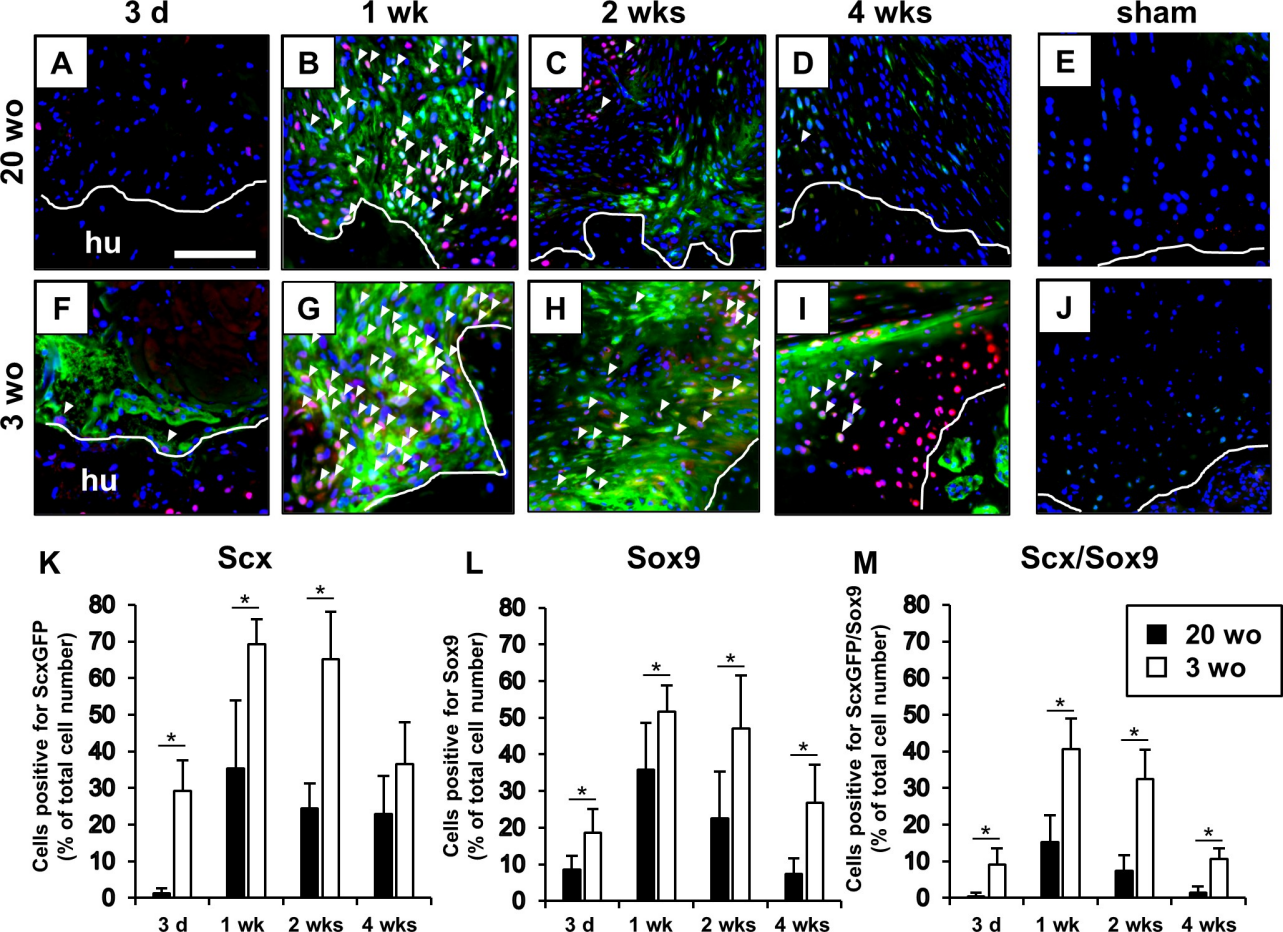
Scx^+^/Sox9^+^ cells in healing after supraspinatus tendon enthesis injury in 20- and 3-week-old mice. (A-J) Microscopic images of the injured enthesis at 3 days (d), and 1, 2, and 4 weeks (wks) and sham-operated control at 4 wks after immunostaining for ScxGFP (green) and Sox9 (red) along with DAPI (blue) in 20 (A-E)- and 3 (F-J)-week-old (wo) mice. Hu, humerus. White arrowheads indicate Scx^+^/Sox9^+^ cells. Solid lines highlight the surface of humeral bone. Scale bar = 100 μm. (K-M) The number of cells positive for Scx, Sox9, and Scx/Sox9 was normalized to the total number of nuclei with DAPI staining at the reparative tissue in 20 and 3 wo mice. All values represent means ± SD. N = 6 for each time point. *p < 0.05.

**Fig 7:**
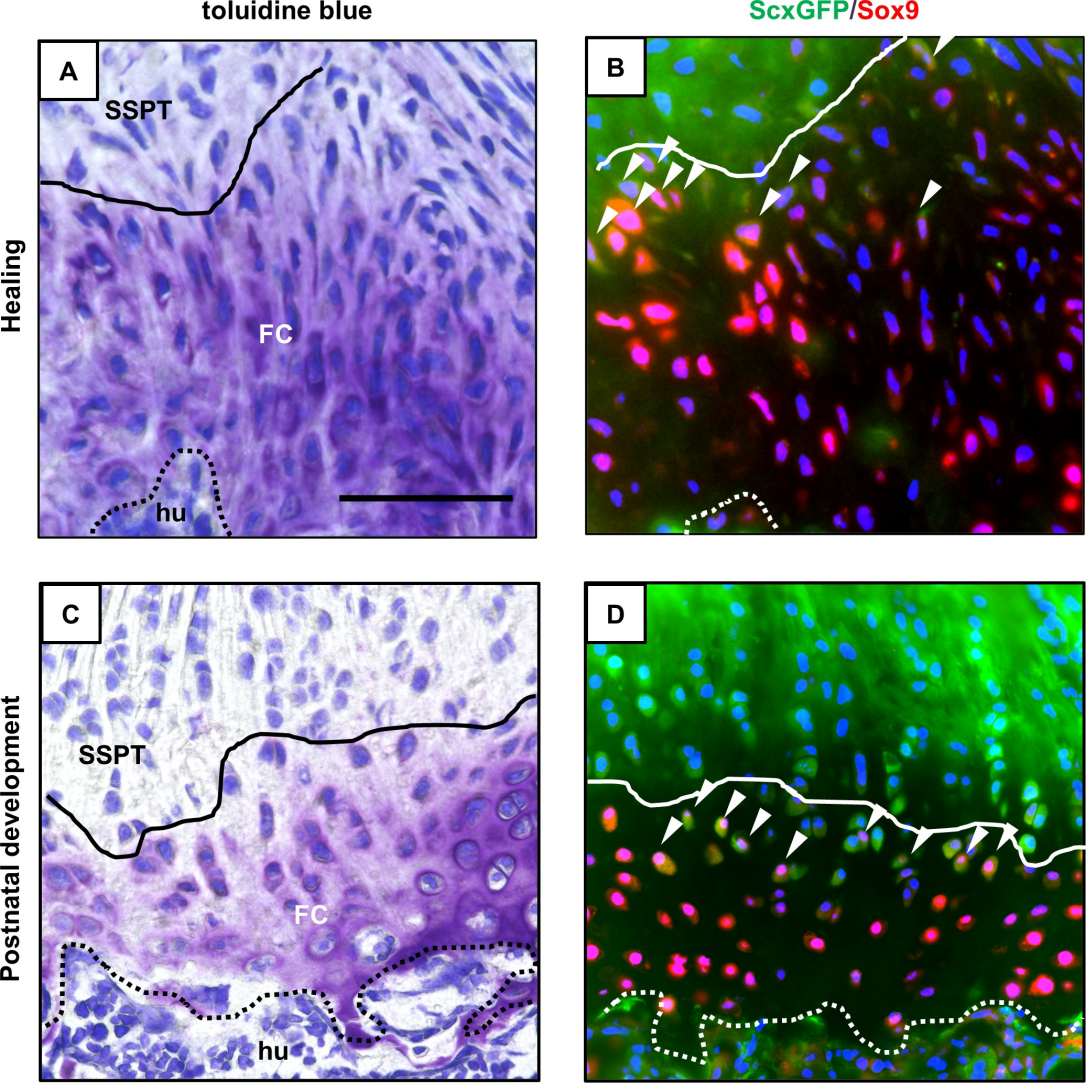
Localization of Scx^+^/Sox9^+^ cells at the entheseal fibrocartilage in postnatal development and in healing. (A-D) Microscopic images of the supraspinatus tendon enthesis at 4 weeks after enthesis injury in 3-week-old mice (A and B) and normal enthesis at 3 weeks of age in postnatal development (C and D). Toluidine blue staining (A and C) and immunostaining of ScxGFP (green) and Sox9 (red) along with DAPI (blue) (B and D) were performed. SSPT, supraspinatus tendon; FC, entheseal fibrocartilage; hu, humerus. White arrowheads indicate Scx^+^/Sox9^+^ cells. Scale bar = 100 μm.

In 20-week-old mice, few Scx^+^/Sox9^+^ cells were observed at the injured site at 3 days after injury (Fig 6A). At 1 week after injury, the cellularity and vascularity were consistently increased from that at 3 days after injury, and scattered spindle-shaped Scx^+^/Sox9^+^ cells were mainly observed near the bone surface within the reparative tissues (Fig 6B). From 1 to 4 weeks after injury, the number of Scx^+^, Sox9^+^, and Scx^+^/Sox9^+^ cells obviously decreased and the Scx^+^/Sox9^+^ cells were rarely seen in reparative tissues at the enthesis, whereas a few Scx^+^ cells were still observed (Fig 6D). In 3-week-old mice, a few Scx^+^/Sox9^+^ cells were observed at the injured site at 3 days after injury (Fig 6F). From 3 days to 1 week after injury, the number of Scx^+^, Sox9^+^, and Scx^+^/Sox9^+^ cells increased, and the majority of the Scx^+^/Sox9^+^ cells were localized near the bone surface within the healing site (Fig 6G). From 1 to 4 weeks after injury, the number of Scx^+^, Sox9^+^, and Scx^+^/Sox9^+^ cells decreased, while the Scx^+^/Sox9^+^ cells were observed at the formed fibrocartilaginous tissue at 4 weeks after injury, in contrast to that in 20-week-old mice (Fig 6I). Between 3- and 20-week-old mice, the percentages of the Scx^+^/Sox9^+^ cells in the reparative tissues were higher in 3-week-old mice at 3 days, 1 week, 2 weeks, and 4 weeks after injury (p < 0.01 for each time point; Fig 6M). Moreover, in 3-week-old mice, the Scx^+^/Sox9^+^ cells were mainly located near the surface of healed fibrocartilaginous tissue at 4 weeks after injury, similar to that in normal entheseal fibrocartilage at 3 weeks of age during postnatal maturation (Fig 7).

### Proliferating Scx-positive cells at the reparative tissue after enthesis injury in 20- and 3-week-old mice

**Fig 8:**
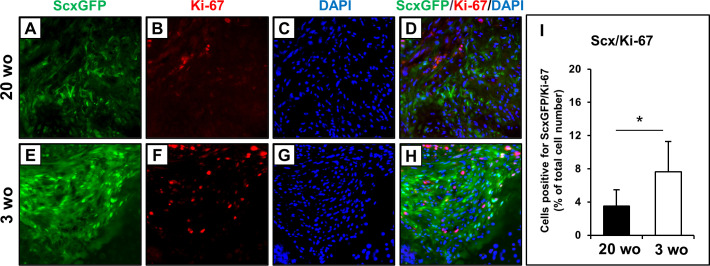
Cell proliferation in the healing site at 1 week after injury. (A-H) Microscopic images of the tendon-bone attachment sites at 1 week after injury, immunostaining for ScxGFP (green, A and E), Ki-67 (red, B and F), DAPI (blue, C and G), and their merged image (D and H) in 20- and 3-week-old (wo) mice. (I) The number of cells positive for ScxGFP/Ki-67 was normalized to the total number of nuclei with DAPI staining at the reparative tissue in 20 and 3 wo mice. All values represent means ± SD. N = 6. *p < 0.05.

To estimate the proliferation of Scx^+^ cells during the healing process in 20- and 3-week-old mice, we performed immunostaining for Ki-67 and ScxGFP at 1 week after enthesis injury. In 20-week-old mice, a small number of Ki-67^+^ cells was observed in the healing site at 1 week after injury, and Scx^+^/Ki-67^+^ cells were rarely seen at the reparative tissue. (Fig 8A–8D). In 3-week-old mice, more Ki-67^+^ and Scx^+^/Ki-67^+^ cells were observed in the reparative tissue than in 20-week-old mice (Fig 8E–8H). The percentage of Scx^+^/Ki-67^+^ cells was significantly higher in 3-week-old mice than in 20-week-old mice (p = 0.04, 3.5% ± 2.0 in 20-week-old, and 7.6% ± 3.6 in 3-week-old mice, respectively; Fig 8I).

## Discussion

Because of poor healing capability, the regeneration of a functional fibrocartilaginous enthesis after injury is a clinical challenge. Thus, identification of enthesis-specific stem or progenitor cells would help in advancing therapeutic approaches for regeneration of functional fibrocartilaginous entheses. Unfortunately, not much is known about entheseal progenitors. In a previous lineage-tracing study using a *ScxGFP* Tg mouse and a *Sox9*^*Cre/+*^*; R26R-tdTomato;ScxGFP* Tg mouse, it was demonstrated that Scx-expressing cells, with a Sox9 expression history, were broadly distributed from cartilage primordia to tendon primordia in a developing enthesis of the Achilles tendon at E14.5 [18]. Furthermore, conditional inactivation of Sox9 in Scx^+^/Sox9^+^ cells using a *ScxCre;Sox9*^*flox/flox*^ mouse model resulted in the defective formation of entheses [18, 19]. These reports suggest that the Scx^+^/Sox9^+^ cells are multipotent progenitors that give rise to tenocytes and ligamentocytes expressing Scx and chondrocytes expressing Sox9, and contribute to the establishment of the enthesis during development.

In the present study, we demonstrate the existence of Scx^+^/Sox9^+^ cells during the postnatal formation of fibrocartilaginous enthesis of the supraspinatus tendon. In addition, prior to formation of the entheseal fibrocartilage layer, the Scx^+^/Sox9^+^ cells were broadly distributed from the tendon insertion site to the tendon substance. These cells were then localized from the developing fibrocartilage surface to the tendon end (Fig 2N). Eventually, they were rarely seen at the enthesis after mature fibrocartilaginous entheses were formed. According to the spatiotemporal localization pattern of Scx^+^/Sox9^+^ cells during the postnatal formation of fibrocartilaginous entheses, it was suggested that the Scx^+^/Sox9^+^ cells might have the ability to differentiate into tenocytes, ligamentocytes, and chondrocytes as in fetal enthesis development. On the other hand, Schwartz et al. used a *Gli1-Cre*^*ERT2*^ mouse model to demonstrate that cells with a Gli1 expression history that originate during the embryonic development eventually populate the mature fibrocartilaginous enthesis [24]. In addition, the ablation of Hedgehog (Hh)-responsive cells during the first week of postnatal development in a *DTA;Gli1-Cre*^*ERT2*^ mouse model resulted in the loss of mineralized fibrocartilage formation. These findings suggest that Hh-responsive cells that have a history of Gli1 expression may play an important role in the formation of mineralized fibrocartilage. Recent lineage tracing experiments using a *Sox9-CreER*^*T2*^*;R26R-tdTomato* mouse have demonstrated that tdTomato-positive cells—indicating embryonic Sox9-lineage cells—are continuously observed at the postnatal development of Achilles entheses from neonate to 2 weeks of age, suggesting that postnatal cells at the Achilles entheses are descendants of embryonic Sox9 lineage cells [25]. Furthermore, it has been shown that stationary entheses, such as the Achilles entheses, develop linearly from embryonic Sox9-positive progenitors that form the postnatal enthesis, and later upregulate the expression of Gli1 [25]. Although the relationship between Scx^+^/Sox9^+^ cells and cells with a history of Gli1 expression is still not clearly understood, in the present study, it was indicated that Scx^+^/Sox9^+^ cells may also play an important role as entheseal progenitor-like cells during the postnatal formation of fibrocartilaginous enthesis of the supraspinatus tendon.

Furthermore, focusing on the healing process after enthesis injury in the present study, we observed that fibrocartilaginous tissue was repaired at the injured bone surface in 3-week-old model mice, although there was no organized 4-layer structure, such as a tidemark or a regular columnar array of chondrocytes observed in normal enthesis. In 20-week-old model mice, the fibrocartilage tissue at the enthesis was not recreated; instead, the fibrovascular scar tissue was formed between the tendon and bone, as previously reported [8–10]. Interestingly, the participation of Scx^+^/Sox9^+^ cells during the healing process after enthesis injury was obviously different between 3- and 20-week-old mice. In 3-week-old mice, some Scx^+^/Sox9^+^ cells were observed at the defect between the torn supraspinatus tendon and bone surface at an early stage; then these cells became widely distributed to the reparative tissue from 1 week to 2 weeks post-injury. With progression of the healing process, the number of Scx^+^/Sox9^+^ cells decreased. On the other hand, a few Scx^+^/Sox9^+^ cells transiently appeared during scar-mediated healing in 20-week-old model mice. These findings strongly suggest that the Scx^+^/Sox9^+^ cells and fibrocartilaginous tissue healing are closely related. Moreover, Scx^+^/Sox9^+^ cells may have been recruited to the healing site because of the response after enthesis injury as they were rarely seen at the enthesis in the sham 20-week-old model mice. Previously, the Scx-expressing cells, which were undetected in the normal tendon in adult mice, were observed at the reparative tissue at 1–2 weeks after injury in a *ScxGFP* Tg mouse patellar tendon window defect model [26]. In addition, it was suggested that the Scx-expressing cells may be differentiated from mesenchymal progenitors expressing α-smooth muscle actin (SMA) [26] or stem cell antigen-1 (Sca-1) [27] that migrated from paratenon, and these cells contribute to favorable tendon healing by producing extracellular matrix to bridge the defect post-injury in a mouse Achilles and patellar tendon healing model. Interestingly, recent lineage tracing experiments using a *Scx-*, *Sox9-Cre*^*ERT2*^, *R26R-tdTomato* mouse have demonstrated that few Scx-lineage cells were observed at the healing enthesis. Meanwhile, some Sox9-lineage cells were observed at the healing unmineralized enthesis fibrocartilage in an adult mouse model of supraspinatus tendon injury and repair, without refreshing of the fibrocartilage layer. These reports indicate that Scx-lineage cells at the enthesis may contribute little to postnatal healing response after enthesis injury under the condition that the fibrocartilage layer is not refreshed in adult mice [28, 29]. In the present study, some Scx^+^, Sox9^+^, and Scx^+^/Sox9^+^ cells were transiently detected during the healing process even in mature mice, by refreshing the fibrocartilage layer. Although it is necessary to consider the difference between the model used in the previous reports [28, 29] and our study, we suggest that recruitment of the Scx^+^ and Scx^+^/Sox9^+^ cells may be associated with removal of the fibrocartilage layer reaching the bone marrow.

Regarding the localization pattern of Scx^+^/Sox9^+^ cells during the healing process in 3-week-old mice, they were widely distributed at the injured site in the early phase, and subsequently localized near the surface of the healed fibrocartilaginous tissue at the enthesis. This spatiotemporal localization pattern of Scx^+^/Sox9^+^ cells was similar to that in postnatal fibrocartilaginous enthesis formation, and this similarity between postnatal fibrocartilage formation and healing indicated that Scx^+^/Sox9^+^ cells may have a role as entheseal progenitor-like cells at injured entheses during the healing process. Recently, Schwartz et al. demonstrated that progenitor-like cells with a history of Gli1 expression, which may contribute to fetal enthesis formation, were transiently detected within the reparative site during scar-mediated healing after supraspinatus tendon enthesis injury in mature mice (7 weeks of age) [21]. In contrast, the progenitor-like cells were maintained during the regenerative healing process in immature model mice (1 week of age) [21] as in our results. It was also demonstrated that more proliferating cells were observed in the regenerative healing in immature mice compared with mature mice in a supraspinatus tendon-bone healing model [21]. Thus, the difference in participation of Scx^+^/Sox9^+^ cells between 3- and 20-week-old mice in the present study may be partly explained by the difference in proliferative capacity of progenitor cells. Collectively, it is suggested that the specific progenitors, including Scx^+^/Sox9^+^ cells, that contribute to fetal tissue formation may also contribute to the regenerative healing process after injury, even in adults. However, the origin, functional role, and differential regulation of Scx^+^/Sox9^+^ cells during the enthesis healing process were not fully elucidated in the present study. Methods to increase the recruitment of Scx^+^/Sox9^+^ cells in reparative entheses may lead to an improvement in healing after enthesis injuries even in adults. We believe that further studies to clarify the regulatory mechanisms involved, particularly concerning the role of Scx^+^/Sox9^+^ cells in the healing process, should provide insights into the development of novel repair-promoting therapeutic strategies to enhance the regenerative healing process following enthesis injury.

## Supporting information

S1 File (DOCX)
