# Supplementary material for: Role of Scx+/Sox9+ cells as potential progenitor cells for postnatal supraspinatus enthesis formation and healing after injury in mice
Source: PLoS One. 2020 Dec 1;15(12):e0242286. doi: 10.1371/journal.pone.0242286 (PMC7707462; doi:10.1371/journal.pone.0242286)
Supplement: S1 File — (DOCX) [file pone.0242286.s001.docx]

Fig 3

Fig 6

20-week-old mice

3-week-old mice

Fig 8

20-week-old mice

3-week-old mice
